# From asymptomatic to critical illness: decoding various clinical stages of COVID-19

**DOI:** 10.3906/sag-2107-137

**Published:** 2021-12-17

**Authors:** İlhami ÇELİK, Recep ÖZTÜRK

**Affiliations:** 1 Department of Infectious Diseases and Clinical Microbiology, Kayseri City Training and Research Hospital, University of Health Sciences, Kayseri Turkey; 2 Department of Infectious Diseases and Clinical Microbiology, Medical School, İstanbul Medipol University, İstanbul Turkey

**Keywords:** COVID-19, clinical features, risk factors, complications, laboratory tests

## Abstract

The clinical course of COVID-19 is variable, with clinical manifestation ranging from 81% mild course to 14% severe course along with 5% critical course in patients. The asymptomatic course is reported to potentially range between 20% and 70% (avg. 33%). A more severe course is seen in the elderly, those with various chronic diseases, and the immunosuppressed, where the case fatality rate is higher in these risk groups. The disease progresses with various symptoms, such as fever, cough, shortness of breath, malaise, myalgia, taste and smell disorders, diarrhea, sore throat, headache, and conjunctivitis. The disease begins with shortness of breath, indicative of lung damage, after an average of 7 to 10 days, and progresses in ARDS, sepsis, and septic shock. Some patients quickly enter shortness of breath, while others gradually develop shortness of breath and chest tightness and burning. The risk factors for a poor prognosis are age, comorbidities, and changes in laboratory tests. Secondary bacterial and fungal infections frequently develop with steroids and immunosuppressants, especially in the intensive care unit. Frequent complications in hospitalized patients include pneumonia (75%), ARDS (15%), acute renal failure (9%), and acute liver injury (19%). An increased incidence of heart damage is observed, including acute heart failure, arrhythmias, and myocarditis. Of the patients hospitalized due to COVID-19, 10%–25% present with prothrombotic coagulopathy, resulting in venous and arterial thromboembolic events. The most common extrapulmonary symptom is neuropsychiatric involvement, frequently accompanied by insomnia, an impediment to remembering, and an altered state of consciousness. During the course of COVID-19, patients undergo some pathological changes (severe lymphopenia, high levels of C-reactive protein, D-dimer, ferritin, etc.) depending on the condition and exposure level of the affected systems as shown by various laboratory tests. The relevant tests are the guiding elements of risk assessment, clinical monitoring, disease severity, and prognosis setting and therapy decision-making processes.

## 1. Introduction

Caused by the agent SARS-CoV-2 and affecting nearly every country globally, the COVID-19 pandemic has resulted in laboratory-verified cases nearing 200 million and over 4,000,000 deaths as of today [1]. 

SARS-CoV-2 is transmitted through the respiratory tract by droplets and contact surfaces contaminated by these droplets (mainly due to hands with the mouth, nose, and eyes). Additionally, it is transmitted airborne by droplet nuclei in clinical procedures (clinical sampling from the respiratory tract) conducted by healthcare providers [2-5].

Every patient may transmit the infection to 2–3 people on average. Transmission is likely in the early period (24–48 h earlier) when there are no symptoms of the disease [2-5]. Some variants developing worldwide during the disease process have increased contagiousness [6].

This paper discussed the varied clinical features and course of COVID-19, providing a further summary of the status of biochemical and hematological tests identified during the disease. 

## 2. Clinical features

The incubation duration of COVID-19 ranges from 2 to 14 days; in many cases, clinical symptoms develop approximately 4 to 5 days after exposure (average incubation time is 4–5.1 days) [7-10]. A study revealed that the symptom onset occurred on average 4 days (3–7 days) after the first positive RT-PCR test [11].

The rate of patients with recent-onset symptoms (shown within 2.2 days) is 2.5%, and 97.5% of those infected were reported to have developed symptoms within 11.5 days [5,7-10]. 

Some publications support that those exposed to the disease without clinical symptoms are higher than expected (20%–70%), and these people are critical regarding transmission [2,5,10]. 

The virus starts to be contagious 24 to 48 h before the symptom onset and remains contagious for 7 to 12 (8) days in mild/moderate cases after the onset of symptoms, where it takes longer than 2 weeks in severe cases; however, positive PCR test results may continue for 6 to 8 weeks [2,5,10].

### 2.1. Severity of the disease and case fatality

COVID-19 is undergoing a critical or severe course ranging from asymptomatic infection to mild or severe respiratory failure; most infections are not severe [2,5,12-16].

The severity index is summarized in Table 1 [5,11,16] when the reported severity information is reviewed.

**Table 1:** Severity of SARS CoV-2 infection, with typical features.

Severity	Indicators	Estimated prevalence
Asymptomatic	Absence of typical or atypical clinical manifestations; no changes in X-ray and/or CT scans	80%
Mild disease	Fever, cough, sore throat, nausea/vomiting, diarrhea, loss of taste or smell but no dyspnea; normal O2 saturation and usually no changes in X-ray and/or CT scans	
Moderate disease	Symptoms of mild disease plus evidence of lower respiratory tract infection (exam and/or imaging (presence of ground-glass opacities and lung consolidation), O2 saturation ≥94% on room air	
Severe disease	Symptoms of moderate disease but O2 saturation <94%, PaO2/FiO2 <300 mmHg, respiratory frequency >30 breaths per minute, or chest imaging showed obvious lesion progression more than 50% within 24–48 h	15%
Critical disease	Symptoms of severe disease but intubated with respiratory failure, septic shock, and/or multiorgan dysfunction; ground-glass opacities (usually bilateral), lung consolidation, pulmonary nodules.	5%

The infection is mild or asymptomatic in 80%–90% of the cases. Only 10% of cases are severe due to dyspnea, hypoxemia, and significant radiological impairment (>50%) of pulmonary parenchyma. About 5% of cases become critical illness and respiratory failure, pneumonia, shock, and multiorgan failure. The most severe cases are accompanied by death due to usual progression to ARDS and multiple organ failure. Generally, this is common in high-risk individuals, such as the elderly and those affected by multiple morbidities. Typically, the affected individuals show varying degrees of dyspnea and radiological symptoms [5,15-23]. In addition, respiratory failure was reported to have developed without a subjective sensation of dyspnea (“silent hypoxemia”) [21]. In these cases, hypocapnia caused by compensatory hyperventilation is an accompanying finding [5].

The spectrum of disease severity and the case fatality rate may vary between countries and even between regions in countries. The estimated fatality rate among infected individuals is relatively low since many COVID-19 infections are asymptomatic, and milder infections are not diagnosed. Based on some analyses, lower and higher rates are also estimated to be between 0.15% and 1%, with significant heterogeneity by reporting location and risk groups [24,25]. The case fatality rate is between 5.8% in Wuhan of China and 0.7% in China [5,17]. There is a higher rate of critical and life-threatening illness among in-patients [19-21]. A study conducted in the US reported the fatality rate among hospitalized patients at 24%, with the fatality rate reported to be 60% in patients using mechanical ventilation in intensive care units [21]. 

In Italy, 12% of all the patients diagnosed with COVID-19 and 16% of hospitalized patients were admitted to intensive care units. The estimated case fatality rate was 7.2% in mid-March 2020 [5,26,27]. On the other hand, South Korea had an estimated mortality rate of 0.9% by mid-March. This might be correlated with different demographic features of the infection; the median age of the infected patients is 64 years in Italy, while the median age in Korea is around 40 years [2,5,15]. 

The variance between several studies is likely to result from distinct patient features (especially elderliness and comorbidities) and/or infection prevalence rates affected by the relative number of diagnostic tests performed on symptomatic individuals [2,17,28-34]. 

### 2.2. Risk factors for severe illness

Symptoms or more severe clinical courses are likely in healthy individuals of any age. However, they have mainly been seen in the elderly or adults with underlying medical comorbidities (cardiovascular diseases, diabetes, chronic lung diseases, etc.) [2,35]. The risk factors for severe diseases are shown in Table 2. Comorbid diseases increase mortality. The rate of preexisting comorbidities was 2.7 in a subgroup of 355 patients deceased from COVID-19 in Italy, where only three patients had no underlying problems [27].

**Table 2:** Proven or potential epidemiological risk factors for severe COVID-19* [5,21,28,37-40].

a) Age > 65
b) Cardiovascular disorder
c) Diabetes mellitus
d) Chronic obstructive pulmonary disease and other pulmonary diseases
e) Cancer (especially hematological malignancies, lung cancer, and metastatic disease)
f) Chronic kidney disease
g) Solid-organ or hematopoietic stem cell transplant
h) Obesity (BMI ≥ 30)
i) Smoking

BMI: Body mass index

Deaths are increasing in older adults. The death rate in elderly patients was considerably higher [5,17,27-36]. A report from the Chinese Center for Disease Control and Prevention found that the case fatality rate was 12% and 15% in a group of people aged 70 to 79 years and people aged 80 years and over, respectively, contrary to the fatality rate of 2.3% in all cohort studies [17]. Similar findings have also been reported in Italy, where mortality rates were 12% for people aged 70–79 and 20% for people aged 80 and over [26]. Sixty-seven percent of the cases in 2449 patients diagnosed with COVID-19 in the US included the population aged ≥45, and similar to the findings in China, the elderly had the highest fatality rate, and the population aged ≥65 years constituted 45% of all the hospitalized patients, 53% of ICU patients, and 80% of deaths. There were no critical care patients or deaths among the population 19 years of age or younger [29].

The rate of severe or life-threatening infections and the need for intensive care were also determined at varying rates in different countries/regions and clinical series [2,5]. In males, a disproportionately high number of deaths have been reported from China, Italy, and the United States [5]. COVID-19 was observed to result in a disproportionately high rate of infection and death, possibly connected with the underlying inequalities in social determinants of health among the black population and Latinos in the United States of America [5].

Symptomatic infection is rare in children; cases are usually mild when they occur, whereas severe cases are reported [5].

Specific laboratory features, such as lymphopenia, elevated inflammatory markers (C-reactive protein [CRP], IL-6, ferritin, etc.), increased liver enzymes, increased lactate dehydrogenase (LDH), high D-dimer (>1 µg/mL), prolonged prothrombin time, high troponin, increased creatine phosphokinase (CPK), and kidney dysfunction, were associated with worse results [5,31-36] (Table 2).

Viral factors (high viral load, viremia) and other genetic factors are associated with the severity of the disease [5].

### 2.3. Asymptomatic infections 

New case series show that asymptomatic infections, which were reported to be low at the initial diagnosis of the disease, are common (20%–70%) [5,6,16,32,33]. A publication evaluating 14 individual studies found that 33% of people infected with SARS-CoV-2 never developed symptoms [41].

Nearly half of the passengers with positive COVID-19 test results as scanned in the cruise ship “Diamond Princess” were asymptomatic during diagnosis; some of these passengers developed symptoms later [5,18]. Asymptomatic cases were found to be higher in especially in the younger population [42,43]. 

Even patients with asymptomatic infections likely have objective clinical abnormalities (low-grade fever, glass opacity in the scanner, irregular shading, etc.). In some asymptomatic cases, different clinical symptoms may subsequently appear [5,18,36,41]. 

### 2.4. Clinical symptoms

COVID-19 often begins with a fever (which develops after other complaints in some cases), followed by tiredness, dry cough, and muscular pain [5,14,29,36]. 

Other cohort studies of the patients with confirmed COVID-19 infection reported similar clinical symptoms [2,5,12,13,36]. Additionally, fever reported at very high rates in initial studies was reported at lower rates in subsequent series; the fever reported in some series was lower than 38 degrees (approximately 20% of the cases) [5,13]. Notably, fever was detected during hospitalization in the first publications, where the fever was detected at a high level (late period) (5, 10, 13). In a study conducted in New York, 31% of patients (>5000 patients) hospitalized for COVID-19 had a fever exceeding 38 degrees [19]. 

The most common severe manifestation of COVID-19 is pneumonia, characterized primarily by fever, cough, shortness of breath, and bilateral infiltrates on chest imaging [5,10,12-14]. In addition, other common features include upper respiratory tract symptoms, myalgia, diarrhea, and olfactory or gustatory disorders. While dyspnea that develops several days after symptom onset provides insight, no specific clinical feature reliably distinguishes COVID-19 from other viral respiratory tract infections [2,5,36]. 

COVID-19 may cause shortness of breath one week after symptoms appear, requiring in-patient treatment in nearly 20% of patients. These symptoms progress into pneumonia in some patients, accompanied by chest tightness, chest pain, and shortness of breath, especially in the elderly and those with chronic health conditions [5,36].

Many studies identifying clinical features of COVID-19 were conducted among the hospitalized populations. As many studies have revealed, clinical symptoms and their frequency are presented in Table 3 [2,5-12,36]. 

**Table 3:** Clinical symptoms identified in several studies [2,5,10-14,36].

Symptoms	Frequency (%)
Fever	43–98
Dyspnea	19–64
Cough	50–82
Fatigue	40–70
Loss of appetite	40–50
Expectoration	14–44
Runny nose	4–24
Sore throat	5–14
Taste and smell dysfunctions	24–60*
Headache	5–34
Diarrhea	2–19
Nausea-vomiting	3–16
Muscle pain	11–36

*Lower in some series (<10%)

### 2.5. Clinical course 

The symptomatic infection may vary from mild to critical during the disease. In most patients (80.9%), infections are mild (with symptoms like the flu) and may be treated at home, 13.8% are severe, leading to severe diseases, such as pneumonia and dyspnea; 4.7% are very severe, resulting in respiratory failure, septic shock, and multiple organ failure. These rates are partly variable in different patient groups [2,5,10-14,36]. 

The disease can progress during a week in some patients without severe initial symptoms. Some mild cases may rapidly become critical. Conditions in moderate cases may worsen more frequently; therefore, this group of patients should be monitored more carefully [5,36].

Patients usually arrive at the hospital on the 4th day from the first symptoms, while mild respiratory distress develops on the 5th day. From the onset of symptoms, hospitalization takes an average of 7 days (4.0–8.0), and respiratory distress occurs on day 8 on average (5.0–13.0), and ARDS on day 9 on average (8.0–14.0). Mechanical ventilation requires an average of about 10.5 days (7.0–14.0) [2,5,12,13,36]. 

The typical course of severe pathology includes overt dyspnea 6 days after the onset of flu-like symptoms, hospitalization after 8 days, and the need for tracheal intubation 10 days after hospitalization [2,5].

 Many of the patients have a good prognosis. The recovery period for mild infection seems to be approximately 2 weeks and 3 to 6 weeks for severe cases. The length of time between clinical onset and death ranges from 2 to 8 weeks [2,5,35]. 

Currently, the number of patients required to be hospitalized is unknown. Approximately 10%–20% of hospitalized patients require intensive care, whereas intubation is required for 3%–10%. The mortality rate for these patients is 2.5% [2,5,10,36].

The disease becomes more severe among the elderly and those suffering from other related conditions (heart disease, hypertension, kidney failure, diabetes, etc.). While the case fatality rate in the population aged under 50 years (with no additional conditions) is low, the risk of fatality is much higher (>10%) in the population aged over 60 years, especially among those aged 70–80 years [2,5,36].

Pediatric patients generally present with mild symptoms. The progression of the illness in pregnant women is similar to that observed in people of the same age group. There is no evidence of intrauterine transmission so far [2,5,36,44].

### 2.6. Severe and critical illness

While most patients with COVID-19 are asymptomatic or have mild SARS-CoV-2 infections, many patients have been admitted to the hospital and ICU. Mortality rates are high during the 2019 Coronavirus Disease (COVID-19) outbreak; the increase in the number of patients requiring an ICU has been overwhelming, jeopardizing most critical care capabilities in hospitals worldwide [45].

Severe and critical cases comprised 14% and 5% of laboratory-confirmed COVID-19 patients, respectively. Clinically, most patients infected with “Severe Acute Respiratory Syndrome Coronavirus 2” (SARS-CoV-2) exhibit no significant symptoms. However, almost 5% of the patients show a severe lung injury or even a “Multiple Organ Dysfunction Syndrome (MODS),” with mortality in intensive care units between 8% and 38%, depending on the country [46].

In a CDC report of 4226 individuals with COVID-19 in the United States between March and February 2020, the overall death rate for patients with COVID-19 was 2.3%, but that was for those requiring treatment and mechanical ventilation due to >50% hypoxemic respiratory failure [30]. 

Our clinical observations indicate that diabetes mellitus (primarily type 2) and underlying heart disease lead to a more severe and life-threatening progression of COVID-19, as well as morbid obesity and autoimmune diseases (Table 4). 

**Table 4:** Risk factors for severe/critical diseases (unpublished data from Kayseri City Training and Research Hospital).

Demographics	Mild/moderaten = 39 (%)	Severe/criticaln = 19 (%)	p
Age (mean ± SD, years)	46.5 ± 15	67.4 ±12	<0.01
Male sex	21 (54)	10 (53)	0.9
COVID-19 compatible chest CT signals	15 (38)	15 (79)	0.05
Chronic obstructive pulmonary disease	24 (36)	6 (32)	0.2
Hypertension	11 (28)	10 (52)	0,07
Diabetes mellitus	16 (19)	9 (50)	<0.01
Coronary artery diseases	15 (18)	5 (26)	0.4
Chronic renal failure	5 (6)	4 (21)	0.03
Body mass index	25 ±3.1	27.1±4.8	0.057

#### 2.6.1. Severe pneumonia

Severe pneumonia should be considered in patients with tachypnea, chest retraction, or inability to eat or drink. In 10% to 20% of severe patients, the airway injury inevitably develops into acute respiratory distress syndrome (ARDS), defined as the oxygen partial pressure (PaO_2_) up to the proportion of inhaled oxygen (FiO_2_) during 8–14 days of illness, the ratio of less than 300 mmHg, as well as resulting noncardiogenic pulmonary edema and mechanical ventilation. ARDS, as the leading cause of respiratory failure, is associated with high morbidity and mortality. Risk factors for developing severe to critical cases are advanced age, underlying comorbidities such as high blood pressure, diabetes, cardiovascular diseases, and cerebrovascular diseases [47]. 

**Figure 1:**
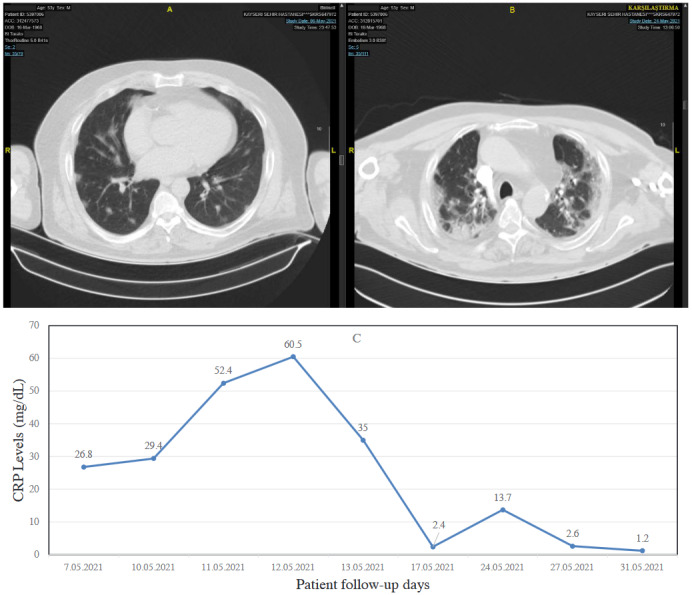
A sample for “severe disease” related to COVID-19. Thoracic CT imaging and CRP course of a 53-year-old male COVID-19 patient with hypertension and coronary artery disease as comorbidities. A) CT imaging at admission had a fever and CRP: 26 mg/dL, commenced dexamethasone at 2 × 8 mg/day. B) Progression of CT imaging on the 9th day of hospitalization. A single dose of 80 mg tocilizumab and dexamethasone 2 × 8mg/day for 10 days were ordered. C) CRP course of the patient.

Severe cases are confirmed if one of the following points is met: (a) shortness of breath, breathing rate ≥ 30 breaths/min; (b) pulse oxygen saturation (SpO_2_) ≤ 93% of the room air at rest; (c) arterial oxygen partial pressure (PaO_2_)/proportion of inhaled oxygen (FiO_2_) ≤ 300 mmHg. At higher altitudes (over 1 km), the PaO_2_/FiO_2_ values should be adjusted based on the equation PaO_2_ / FiO_2_ × [atmospheric pressure (mmHg) / 760]; (d) patients with >50% lesion progression within 24 to 48 h on lung imaging [48]. The chest CT and the evolution of the CRP of a severe case of pneumonia are illustrated in Figures 1 a–1c. 

The mean time from symptom onset to pneumonia development is about 5 days. In the meantime, from symptom onset to the commencement of severe hypoxemia and being hospitalized in the ICU is about 7–12 days [29].

Most patients have bilateral opacities on chest X-ray and tomography. The most common findings on tomography are ground-glass opacities and consolidation. In patients admitted to the ICU, acute respiratory distress syndrome (ARDS) with acute hypoxemic respiratory failure—sometimes with severe hypercapnia—is the most common complication (60%–70%), followed by shock (30%), myocardial dysfunction (20%–30%) and acute kidney injury (10%–30%). Elderly patients may develop hypoxemia without respiratory distress. Arrhythmia is observed in nearly half of the patients [49]. Some notable laboratory parameters for mild/moderate and severe/critical patients that were followed in our hospital are shown in Table 5.

**Table 5:** Laboratory features of the patients at admission time associated with severe/critical COVID-19 (unpublished data from the Kayseri City Hospital).

Variables (unit, normal values)	Patients		p
	Mild/moderate(n = 39)	Severe/critical(n = 19)	
White blood cell count, ×10³μL (4.5–10)	5.7 (4.8–7.3)	8.8 (6.5–10.3)	0.002
Neutrophils, x10³μL (1.8–7.5)	3.5 (2.6–5.1)	6.3 (3.9–9.1)	0.001
Lymphocytes, x10³μL(1.1–3.2)	1.6(1.3–1.9)	1.1 (0.8–1.7)	0.021
Lactate dehydrogenase, U/L(135–214)	210 (189–271.0)	325.5 (233.0–376.0)	0.004
Ferritin, µg/L(30–400)	123.0 (79.0–228.0)	473.0 (206.0–902.0)	0.001
C–Reactive Protein, mg/dL(0–5)	6.9 (1.7–30.6)	93.0 (47.0–137.0)	<0.001
D–dimer, µg/L (0–500)	390.0 (290.0–670.0)	1010.0 (685.0–580.0)	0.002

### 2.7. Critical COVID-19

**Figure 2:**
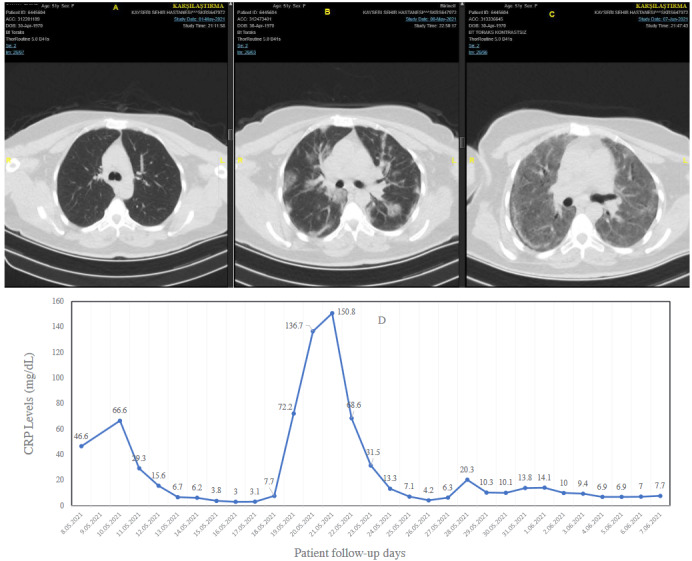
A sample for critical disease associated with COVID-19. Thoracic CT imaging and CRP course for 51-year-old women COVID19 patient with previously undiagnosed type II diabetes mellitus. A) Thoracic computed tomography at admission. B) Thoracic CT imaging on day eight. C) Thoracic CT imaging on the 37th day. D) Patient’s CRP course. Low-dose tocilizumab (100 mg, 100 mg, every other day at first CRP peak, and 80 mg, and 80 mg one dose at second CRP peak, every other day) four times (a total of 360 mg) and 250 mg three times for 3 consecutive days methylprednisolone and 40 mg/day for 20 days had been administered.

In critical cases, patients complained of respiratory failure (severe dyspnea, shortness of breath > 30/min, tachypnea, hypoxia), fever, reduced blood oxygen saturation of less than or equal to 93%, and pulmonary filtrates, shock, and multiorgan dysfunction, or even failure. Research has also shown that, under severe conditions, patients show signs of impaired breathing, wet rattles, dull percussion, and a decrease in tactile tremor. Apart from respiratory problems, the disease also causes gastrointestinal issues like diarrhea. Laboratory tests of a complete blood count may show an average or reduced white blood count or a reduced lymphocyte count at the beginning of the onset of the disease. Patients also have higher levels of C-reactive protein. The chest CT series and the progression of the CRP of a critical case are illustrated in Figures 2a–2d.

Critical cases are recognized when one of the following points is met: (a) respiratory failure occurs, and mechanical ventilation is required; (b) shock occurs; (c) complicated with other organ failures that require monitoring and treatment in the intensive care unit (ICU); individuals who have acute respiratory failure, septic shock, and/or multiple organ dysfunction. Patients with severe COVID-19 illness may become seriously ill due to the onset of “acute respiratory distress syndrome” (ARDS), which tends to occur approximately one week after symptoms appear [50]. 

### 2. 8. Acute respiratory distress syndrome (ARDS)

In patients with respiratory manifestations of SARS-CoV-2 infection, hypoxemic respiratory failure may occur. After the onset of shortness of breath, patients initially appear to have good lung compliance and poor lung elasticity, and an inadequate response to positive expiratory pressure (PEP); some patients may then progress to an acute respiratory distress syndrome, which is characterized by high elasticity and low compliance, as well as a high PEP response. In addition, it appears that endothelial damage is occurring, resulting in disruption of pulmonary vasoregulation and inadequate ventilation and perfusion. ICU admission and mechanical ventilation rates vary from report to report, possibly due to differences in age, admission threshold, mechanical ventilation, and the timing of the study conducted with the time of the pandemic [51].

Oxygenation disorder in adults:

· Mild ARDS: 200 mmHg <PaO_2_/FiO_2_a ≤ 300 mmHg (PEEP or CPAP ≥ 5 cmH2O).

· Moderate ARDS: 100 mmHg <PaO_2_ / FiO_2_ ≤ 200 mmHg (≥ 5 cmH2O with PEEP).

· Severe ARDS: PaO_2_ / FiO_2_ ≤ 100 mmHg (with PEEP ≥ 5 cmH2O) (52).

### 2. 9. Sepsis and septic shock

**Figure 3:**
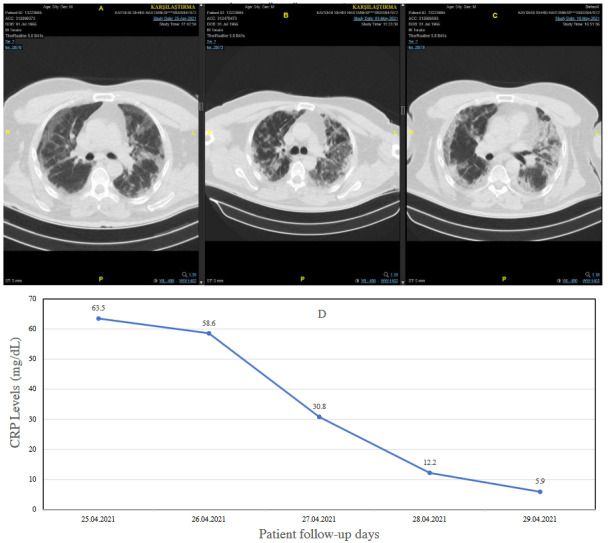
A sample for sepsis/septic shock and MODS associated with COVID-19. A 54-year-old male with type 2 diabetes complained of shortness of breath (SaO2: 77) and weakness and was monitored in the ICU. A) Chest CT at the time of admission (CT) showed a typical ground glass image that corresponded to a COVID-19 peripheral weighted bilateral multifocal patchy. B) Chest CT on day nine at hospitalization. C) Chest CT on day 23 and out of the hospital with an oxygen condenser. D) CRP course of the patient.

**Figure 4:**
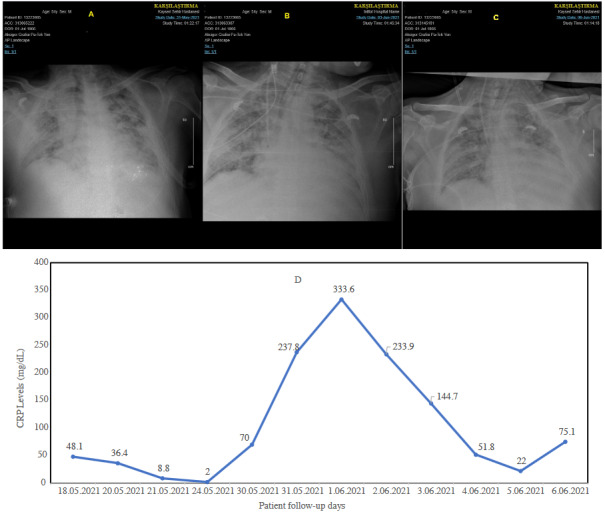
The same patient (a 54-year-old male) was hospitalized again after a 12-day discharge with severe signs of ARD. A) On the day of admission, he vented mechanically. B) 3rd day of re-admission, and C) 6th day of re-admission, D) The patient’s CRP course lost 43rd day because of septic shock and multiple organ failure.

Patients with COVID-19 and sepsis are thought to be the most critical. The accompanying multiorgan dysfunction results from a deregulated host response to infection. Signs of organ dysfunction include severe dyspnea, low oxygen saturation, decreased urinary output, tachycardia, hypotension, cold extremities, skin patches, and impaired mental status. Laboratory evidence of other homeostatic disorders includes acidosis, lactate elevation, hyperbilirubinemia, thrombocytopenia, and signs of coagulopathy. Blood pressure of patients with septic shock is continuously decreased despite the volume of resuscitation. They may also have an associated serum lactate level of> 2 mmol / L [53]. The chest CT series and the CRP progress of a MODS and ARDS are shown in Figures 3a–3d and Figures 4a–4d, respectively. 

### 2. 10. Complications

**Figure 5:**
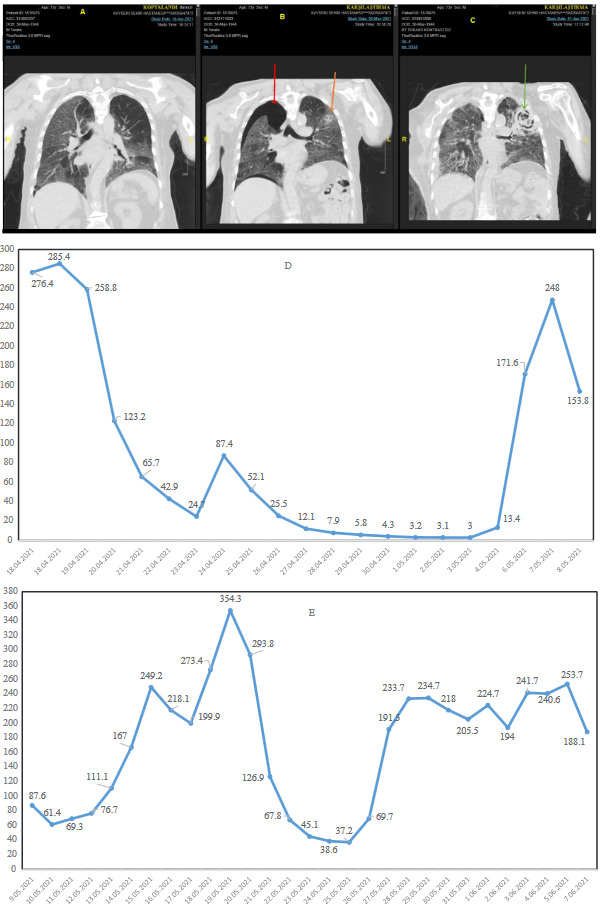
A 73-year-old male patient was admitted to our hospital for a critical illness related to COVID-19. A) The chest computed tomography results were compatible with CORADS-5. B) Pneumothorax (red line) and invasive fungal infection are seen as a mass with a peripheral halo (orange line) of ground-glass opacification on day 20th of hospitalization in the ICU. C) Reversed halo sign (RHS) in the left upper lobe (green line) with adjacent ground-glass opacities at the 43rd day of hospitalization in ICU. D & E) Patient’s CRP course. Aspergillus niger was recovered from bronchoalveolar lavage fluid sample, and galactomannan in bronchoalveolar lavage fluid antigen test was reported as “positive: 7.19” (0.16–0.2 index).

Complications of COVID-19 included pneumonia, acute respiratory distress syndrome, heart damage, arrhythmia, septic shock, liver dysfunction, acute kidney injury (AKI), and multiple organ failure. Approximately 5% of COVID-19 patients and 20% of hospitalized patients present severe symptoms requiring critical care. The most common complications in hospitalized patients are pneumonia (75%), ARDS (15%), acute kidney injury [AKI (9%)], and acute liver injury (19%). A growing quantity of heart damage has been found, including increased troponin levels, acute heart failure, arrhythmias, and myocarditis. Of hospitalized COVID-19 patients, 10%–25% develop prothrombotic coagulopathy, which results in venous and arterial thromboembolic events. Neurological manifestations include altered consciousness and stroke [48]. A variety of disease-related complications and their physio-pathological mechanisms were summarized in Table 6. The thoracic CT series and CRP progression of a case complicated by pneumothorax and invasive pulmonary aspergillosis are shown in Figures 5a–5e.

**Table 6:** Complications of severe COVID-19 infection [54].

Complication	Inflammatory response	Structural abnormalities	Clinical outcome
Abnormal coagulation and thrombosis	LymphopeniaThrombocytopenia­ IL-6, CRPD-dimmer	Venous thrombosisIntravascular coagulopathy,Myocardial injuryCerebral infarction	Deep vein thrombosisDICMyocardial infarctionStroke
Acute cardiovascular syndrome (ACovSC)	­Troponin¯ST or ­STArrhythmias	Wall motion abnormalities,De novo systolic dysfunctionMyocarditis or myocardiopathy	Heart failurePericardial effusionAcute coronary artery disease.
Acute respiratory distress syndrome (ARDS)	Alveolar damage Influx of inflammatory cellsProtein exudation	Pulmonary edemaPneumonia, Pleural effusionSecondary bacterial infection	Acute dyspneaSevere hypoxiaRespiratory failure
Cytokine storm syndrome (CSS)	Macrophage activation­­Cytokine release­Thrombin ¯Anticoagulants	Activation of coagulation pathwaysWidespread microthrombi, ­Vascular permeability	DICShockMultiorgan failure

### 2. 11. Viral and bacterial coinfection and secondary infection in patients with COVID-19

According to current estimates, there is generally a low rate of new, real viral coinfections at the beginning of COVID-19. Reports of high coinfection rates are expected to be tempered by the limitations of current diagnostic methods. Nevertheless, more data collection is warranted, and more information about COVID-19 coinfections will provide better guidance for effective antimicrobial treatment, whether SARS-CoV-2 coinfections or genuine coinfections [55]. 

In a multicenter study by Russell et al. [56], 1107 patients/48,902 patients (2.26%) were identified as having COVID-19-related infections (positive blood, sputum, or deep breathing cultures). For microbiologically confirmed COVID-19-associated infections where sampling time was known, 762 (70.6%) of the 1080 infections were secondary and occurred more than 2 days after hospitalization.

Most culture-positive respiratory samples represented secondary infections (919 [4.9%] of 1082 sputum and deep respiratory cultures). In lower respiratory tract coinfections (within 2 days of admission), *Staphylococcus aureus* (17.8%), *Haemophilus influenzae* (12.7%), and *Pseudomonas aeruginosa *(9.3%) were most frequently identified in sputum. *Streptococcus pneumoniae* was infrequently cultured (4·2%). *Staphylococcus aureus* was the predominant organism (31.1%). *S. aureus* was the common cause of secondary lower respiratory tract infections (sputum: 12.6%; deep respiratory: 10.5%), but most organisms were gram-negative, including *Escherichia coli* (14.5%), *Pseudomonas aeruginosa* (12.5%), *Klebsiella pneumoniae *(11.8%), *Klebsiella aerogenes *(6.2%), and *Citrobacter koseri* (4.0%) [57]. 

### 2. 12. Extrapulmonary involvement of COVID-19

#### 2. 12. 1. Neurologic involvement

In patients with severe or critical comorbidities, neurological complications occur frequently. Central and peripheral nervous systems (CNS and PNS) may be affected during COVID-19. CNS symptoms include headache, and decreased reactivity are initial indicators of potential neurological impairment; anosmia, hyposmia, hypogeum, and dysgeusia are also common early symptoms of coronavirus disease. Headache is a commonly occurring symptom. The most severe neurological manifestation, sensory impairment (agitation, delirium, and coma), is caused by hypoxia and metabolic abnormalities [58].

Cerebrovascular disease, particularly large-vessel ischemic strokes, and less frequently cerebral venous thrombosis, intracerebral hemorrhage, and subarachnoid hemorrhage, usually occur as part of a thrombotic state induced by viral attachment ACE2 receptors in the endothelium. In addition, acute hemorrhagic necrotizing encephalopathy is associated with cytokine storms [59].

Additionally, individual cases of convulsions, encephalopathy, meningitis, encephalitis, and myelitis have been reported. However, in the case of COVID-19, neurological diseases affecting PNS and muscles are less common. They include Guillain–Barré syndrome, Miller Fisher syndrome, cranial polyneuritis, and rare cases of viral myopathy accompanied by rhabdomyolysis [60].

#### 2. 12. 2. Psychiatric involvement

There has been an increase in anxiety, mood, sleep, substance use, and posttraumatic stress disorders after epidemics. In addition, anxiety related to illness, social and economic issues, and quarantine may cause more stress and depressive symptoms in individuals during the pandemics. In particular, people who contract the disease, those at heightened risk for it (including the elderly, people with compromised immune function, and those living or receiving care in congregate settings), and people with preexisting medical, psychiatric, or substance use problems are at increased risk for adverse psychosocial outcomes [61].

#### 2. 12. 3. Cutaneous involvement

In an international study, the most common dermatological morphologies were found in 171 laboratory-confirmed cases of COVID-19, including morbilliform (22%), perniform (18%), urticaria (16%), macular erythema (13%), vesicular (11%), papulosquamous (9.9%), and retiform purpura (6.4%) [62].

#### 2. 12. 4. Cardiac involvement

Coupled with the high inflammatory load associated with cytokine release, COVID-19 can trigger vascular inflammation, acute myocardial injury, myocarditis, arrhythmias, venous thromboembolism, metabolic syndrome, and Kawasaki disease. The most frequently reported cardiovascular complications of COVID-19 include acute myocardial injury, myocarditis, myocardial infarction, heart failure, cardiomyopathy, arrhythmias, and venous thromboembolic events [63]. 

A case series evaluated cardiovascular events included 84 patients from Morocco; 50 had a pulmonary embolism (59.52%); three of them were massive. Twelve patients had a myocardial infarction (14.28%), ten had pericarditis (11.9%), and three developed myocarditis (3.57%). In addition, six patients had ischemia (7.14%), including two patients with Leriche syndrome (in one case associated with segmental pulmonary embolism), three patients with lower limb ischemia, and only 1 case of upper limb ischemia. There were also 2 cases of ischemic stroke (2.38%) and 1 case of congestive heart failure (1.19%) [64]. 

#### 2. 12. 5. Renal involvement

A recently published study assessed over 1000 cases of COVID-19 in China and found that the prevalence of acute kidney injury (ARI) in these patients was 1.6% (2). Another study conducted at a university hospital in Wuhan City in China evaluated 701 COVID-19 patients and found that the overall prevalence of ARI caused by COVID-19 was 3.2% (3). Another interesting neurological conclusion was that 43.9% of patients had proteinuria when admitted, and 26.7% had hematuria. Rhabdomyolysis is commonly observed in COVID-19. It can lead to high levels of serum phosphokinase creatinine (more than five times the upper bound of the ordinary) and could be another critical factor in ARI development [65]. 

#### 2. 12. 6. Endocrine involvement

To date, evidence indicates that COVID-19 can induce a functional pituitary gland by direct and indirect effects on the hypothalamus-pituitary axis, resulting in an inappropriate response to adrenal stress. Furthermore, there are several reports of potential immune damage to the thyroid gland leading to subacute thyroiditis. COVID-19 has been implicated in high blood glucose in known diabetics and the discovery of insulin resistance in previously undiagnosed people. It has also been shown that COVID-19 triggers type 1 diabetes associated with ketosis. The presence of the virus in semen currently has no apparent clinical significance [66].

#### 2. 12. 7. Gastrointestinal features

The prevalence of gastrointestinal symptoms at the time of diagnosis differs from study to study, ranging from 2% to 57%. For example, in a metaanalysis of 35 studies in China, mainly conducted in the first phase of the epidemic, the clinical characteristics of 6686 COVID-19 patients were analyzed, finding a combined prevalence of gastrointestinal symptoms by 15%. In addition, transient elevations in serum aminotransferases and altered markers of liver function and bilirubin have been observed in up to 58% of patients with severe COVID-19. However, the underlying mechanisms and possible complications are still poorly understood [67]. 

#### 2.12.8. Ocular involvement

In studies that reported details of observed eye symptoms, the most common eye symptoms were dry eye or foreign body sensation (16%), redness (13.3%), tearing (12.8%), itching (12.6%), eye pain (9.6%), and discharge (8.8%) [68].

## 3. Laboratory tests 

During the course of COVID-19, patients have various pathological changes as indicated by laboratory tests. The publications on these changes in laboratory tests increased over time, and the results of different studies were initially assessed and analyzed [2,5,34-36].

The disorders diagnosed upon hematological, immunological, and biochemical tests are usually diagnosed in hospitalized patients. The summary information on the tests used in this context is given in Table 7. 

**Table 7:** Common laboratory findings among hospitalized patients with COVID-19* [2,5,34-36].

Tests	Changes and interpretations
Hematological parameters (including hemostasis and coagulation)	
Complete blood count (hemogram)	Hemoglobin (usually normal, may be decreased in severe cases)Leukocyte (normal, decreased, increased (in ICU cases)) Lymphopenia (in most cases), <800 μL in severe casesEosinopenia (in most cases)Thrombocyte (normal, increased, slightly lower in severe cases; thrombocytopenia is linked to the disease severity and the mortality risk.
CD4 and CD8	Decreased (in most cases)
PT and aPTT	Normal, slightly prolonged (in severe cases, especially in ICU cases); identification of coagulopathy
Fibrinogen	Decreased; identification of continued coagulopathy
D-dimer	Increased (in severe cases; in nonsurvivors); >1000 ng/mL: in severe cases; identification of continued consumption and thrombotic coagulopathy
Inflammatory Markers	
CRP	Increased (Higher in severe cases); > 10 × the upper limit of normal (N: <8 mg/L)
Erythrocyte sedimentation rate	Normal, increased
Ferritin	Increased (in severe cases); >500 µg/L (N: 10/30–200/300 µg/L)
Procalcitonin	Normal, Increased (In severe cases, secondary to bacterial pneumonia in ICU patients)
Il-6	Increased (according to severity: >critical>severe>mild; cytokine storm)
Biochemistry tests	
Glucose	Increased (in severe cases); a marker of metabolic balance
BUN and creatinine	Increased (in severe cases); a marker of renal damage and failure
Electrolytes	Hyponatremia, hypokalemia (hyperkalemia in some patients), Hypocalcemia; a marker of metabolic balance
Albumin	Decreased (in severe cases; a marker of liver failure; associated with increased mortality)
ALT and AST	Increased (especially in ICU cases; usually as a result of cytokine storm or drug-induced liver injury)
Total Bilirubin	Increased (in severe cases and/or prolonged cases of hospitalization); as a result of liver injury
Lactate dehydrogenase	Increased (in severe cases, in ICU cases, in ARDS cases); >245 U /L in severe cases (N: 110–210 u/L); a marker of lung injury and tissue damage
Creatine phosphokinase	Increased in severe cases; > 2 × the upper limit of normal (N: 40–150 U/L)
Troponin T/I	Increased (in severe cases), > 2 x the upper limit of normal (N: 0–9/14 ng/L)
B-type natriuretic peptide (BNP/NT-proBNP):	Increased (in severe/critical cases)

*Of these general laboratory features, the ones identified by us clinically are discussed in relevant sections above.

Laboratory tests guide severity and risk assessment, clinical monitoring, and therapy decision-making processes [34,35].

As laboratory findings in hospitalized patients, some values (CRP, sedimentation rate, LDH, ferritin, etc.) increase at varying levels along with low lymphocyte count and high liver enzymes [2,5,36]. 

The most common laboratory findings in COVID-19 patients are elevated CRP levels, decreased albumin levels, increased ESR, lymphopenia, eosinopenia, increased IL-6, and increased LDH.

As revealed by several studies, the most common laboratory abnormalities reported at admission among hospitalized pneumonia patients include leukopenia (9%–34%) or leukocytosis (24%–30%), lymphopenia (63%–83%), thrombocytopenia (34%), and elevated aminotransferase (37%) and lactate dehydrogenase [2,5,10,12-14,34-36]. 

Increased inflammation indices, including nonincreased procalcitonin and increased CRP levels, are associated with clinical severity. At admission, many patients with pneumonia have normal serum procalcitonin levels; however, this level could likely be higher in patients who need care in ICUs [5,9,29-31]. High CRP levels were found in hypoxemic COVID patients, and a further correlation was observed between CRP and mortality risk [2,5,34-36]. Troponin appears to be a strong prognostic marker of mortality. Additionally, D-dimer and ferritin levels were notably higher in hospitalized patients in general. If necessary, patients with increased CRP, lymphopenia and increased LDH should be followed closely and transferred to ICUs. Various laboratory features, including high D-dimer levels and more severe lymphopenia, were associated with critical disease and mortality [2,5,14,34-3]. 

In conclusion, COVID-19 is a novel viral disease with the potential to affect almost every system and a rich clinical spectrum, which results in varied complications and is particularly severe and fatal in the elderly or those with comorbidities, leading to pathological changes in various laboratory tests.
